# A Study of High-Resolution Ultrasound and Magnetic Resonance Imaging Findings in Shoulder Joint Pain at a Tertiary Care Hospital in Central India

**DOI:** 10.7759/cureus.66518

**Published:** 2024-08-09

**Authors:** Bhagyasri Nunna, Pratapsingh Parihar, Pankaj Nagtode, Nikita Bora, Neha D Shetty, Rishabh Dhabalia

**Affiliations:** 1 Radiodiagnosis, Jawaharlal Nehru Medical College, Datta Meghe Institute of Higher Education and Research, Wardha, IND

**Keywords:** diagnostic efficacy, biceps tendinopathy, rotator cuff injuries, shoulder joint pain, magnetic resonance imaging, high-resolution ultrasound

## Abstract

Objective

This study aims to investigate the diagnostic efficacy of high-resolution ultrasound (USG) and magnetic resonance imaging (MRI) in patients with shoulder joint pain at a tertiary care hospital in Central India.

Methods

This cross-sectional study was conducted at Acharya Vinoba Bhave Rural Hospital from 2021 to 2024. The study population consisted of patients with shoulder pain, without fractures, who were evaluated using USG and MRI. Participants with infective arthritis, rheumatoid arthritis, previous shoulder surgery, or contraindications for MRI were excluded. Data were recorded and analyzed using Microsoft Excel (Microsoft Corporation, Redmond, Washington) and R 4.2.0 software (The R Foundation, Vienna, Austria). Sensitivity, specificity, and receiver operating characteristic (ROC) curves were used to compare the diagnostic performance of USG and MRI.

Results

A total of 80 patients were included, with 49 (61%) males and 31 (39%) females. The MRI findings showed supraspinatus partial tears in 44 (55%) cases, complete tears in 10 (12.5%), and various other shoulder pathologies. USG detected supraspinatus partial tears in 16 (19.5%) and complete tears in seven (8.8%). Kappa statistics indicated moderate to high agreement between USG and MRI for several pathologies, with near-perfect agreement for complete tears.

Conclusion

High-resolution USG is a valuable tool for the initial assessment of shoulder joint pain, providing reliable diagnostic information with high agreement levels with MRI for complete tears and certain shoulder conditions. MRI remains indispensable for comprehensive evaluation, particularly for partial tears and complex pathologies.

## Introduction

Shoulder pain is a prevalent musculoskeletal complaint, affecting approximately 18%-26% of the general population at some point in their lives [1]. It is one of the leading causes of musculoskeletal disability, significantly impacting individuals' daily activities and quality of life. The shoulder joint's complex anatomy and wide range of motion make it particularly vulnerable to various injuries and conditions, such as rotator cuff tears, tendinopathies, bursitis, and degenerative joint diseases [2]. These conditions can lead to chronic pain, reduced mobility, and functional impairment if not accurately diagnosed and effectively treated [3]. Accurate and timely diagnosis of shoulder pathologies is crucial for guiding appropriate management and therapeutic interventions.

Magnetic resonance imaging (MRI) is widely regarded as the gold standard for evaluating soft tissue injuries and joint disorders due to its superior contrast resolution and ability to provide detailed images of the shoulder's internal structures [4]. MRI can effectively identify rotator cuff tears, labral injuries, tendinopathies, and other intra-articular and peri-articular pathologies. However, MRI is expensive, less accessible in rural and resource-limited settings, and may not be suitable for all patients due to contraindications such as the presence of metal implants, pacemakers, or claustrophobia [5].

High-resolution ultrasound (USG) has emerged as a valuable alternative for shoulder imaging, offering several advantages, including cost-effectiveness, accessibility, and the ability to perform real-time dynamic assessments. USG is particularly useful in detecting rotator cuff tears, tendinopathies, bursitis, and other soft tissue abnormalities [6]. It allows for the visualization of tendon movement and can be used to guide therapeutic interventions such as injections. However, the diagnostic accuracy of USG compared to MRI, especially in detecting partial tears and subtle pathologies, remains a subject of ongoing research [7]. Several studies have demonstrated the efficacy of USG in diagnosing shoulder pathologies, with reported sensitivity and specificity rates comparable to those of MRI for certain conditions. However, operator dependency and variability in the USG technique can affect the accuracy and reliability of the results [8]. Therefore, there is a need for further research to validate these findings and determine the feasibility of using USG as a primary diagnostic tool for shoulder pain, particularly in settings where MRI may not be readily available [9].

This study aims to evaluate the diagnostic efficacy of high-resolution USG compared to MRI in patients presenting with shoulder joint pain at a tertiary care hospital in Central India. By assessing the sensitivity, specificity, and agreement levels between these imaging modalities, the study seeks to determine the viability of USG as a primary diagnostic tool in the initial assessment of shoulder pain. This research is particularly relevant for resource-limited settings, where access to advanced imaging modalities such as MRI is often limited. Additionally, the study aims to identify the prevalence of various shoulder pathologies in the study population, providing valuable insights into the common causes of shoulder pain in this region.

## Materials and methods

Study design, population, and place of study

This study employed a cross-sectional design to investigate high-resolution USG and MRI findings in patients with shoulder joint pain. The research was conducted at Acharya Vinoba Bhave Rural Hospital (India) from 2021 to 2024. The study population consisted of patients recruited from those attending rural hospitals or clinics serving as primary healthcare centers for the surrounding rural community. The research was conducted in the Department of Radiology at Datta Meghe Institute of Higher Education and Research (DMIHER).

Participants

Participants eligible for inclusion in this study met specific criteria, including a history of shoulder pain affecting one or both joints, with or without prior trauma, that did not involve any fractures around the shoulder joint. Additionally, individuals with suspected rotator cuff injuries, encompassing partial and complete tears, and those presenting with injuries to the biceps tendon or calcific tendinitis were considered for enrollment. These criteria ensured that the study focused on patients experiencing shoulder joint-related symptoms potentially indicative of underlying pathologies suitable for assessment through ultrasound and MRI imaging techniques.

Conversely, participants were excluded from the study if they presented with identified cases of infective arthritis or rheumatoid arthritis, conditions that could confound the interpretation of imaging findings related to shoulder pain. Additionally, individuals with a history of previous shoulder surgery or those currently possessing a shoulder prosthesis were excluded due to potential alterations in joint anatomy that could impact imaging outcomes. Patients with pacemakers, metal implants, foreign bodies in their eyes, or who reported suffering from claustrophobia were also excluded to ensure the safety and feasibility of undergoing MRI procedures. These exclusion criteria were crucial for maintaining the integrity of the study's imaging assessments and ensuring participant safety throughout the research process.

Procedure

**Figure 1:**
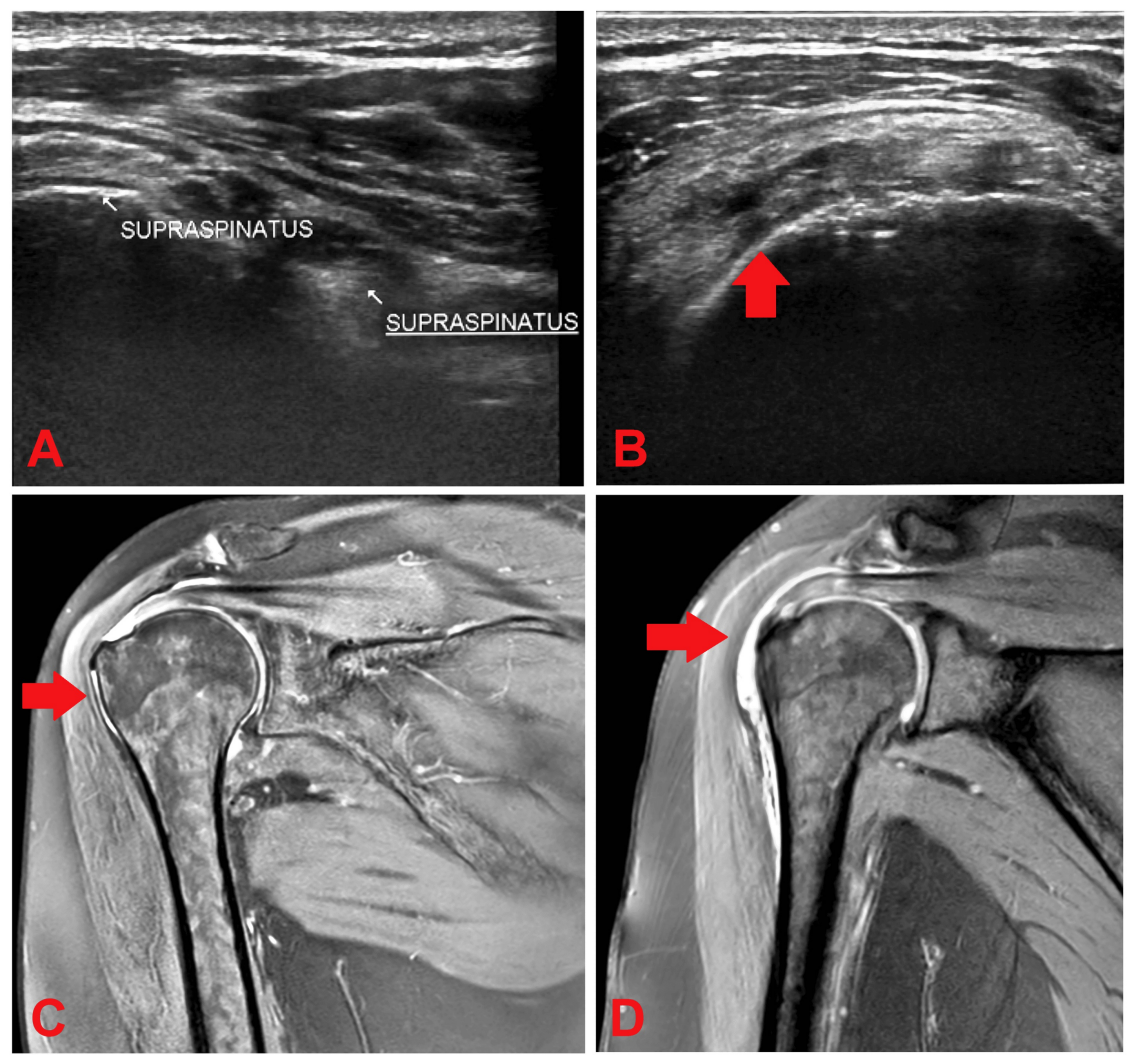
(A) USG image of supraspinatus complete tear; (B) USG image of supraspinatus partial tear; (C) MRI image of supraspinatus complete tear; (D) MRI image of supraspinatus partial tear Image credit: Dr. Bhagyasri Nunna USG: high-resolution ultrasound; MRI: magnetic resonance imaging

All eligible participants meeting the inclusion criteria were actively recruited for the study. Explicit informed consent was diligently obtained from each participant before any procedures. Initially, participants underwent high-resolution USG using the Aloka Hitachi USG machine Arietta S70 (Tokyo, Japan) [10], equipped with a linear frequency probe ranging from 12 to 18 MHz and complemented by color Doppler imaging capabilities. Participants displaying positive findings or suspicious pathologies during ultrasonography were subsequently scheduled for MRI, conducted on a Philips 3 Tesla MRI machine (Amsterdam, Netherlands). Patients' diagnostic images are shown in Figure 1. This multimodal approach facilitated the assessment of agreement between ultrasound and MRI findings. Data collected were meticulously recorded and formatted using Microsoft Excel (Microsoft Corporation, Redmond, Washington). A comprehensive master chart was created, the foundation for subsequent statistical analyses.

Data management

Study materials were considered confidential and securely stored, with access restricted to the principal investigator. This measure ensured the security and confidentiality of participant data and study-related documents, maintaining the integrity and privacy of the collected information.

Ethics and consent

The Institutional Ethical Committee of DMIHER approved the research protocol in a meeting held on November 7, 2022, with reference number DMIMS(DU)/IEC/2022/28. All participants were thoroughly informed about the research, and written and verbal informed consent was obtained from each participant before any intervention. The study protocol has been published in F1000Research [11].

Statistical analysis

Statistical analysis was carried out using R 4.2.0 software (The R Foundation, Vienna, Austria). All relevant statistical data were tabulated on a spreadsheet using Microsoft Excel. Statistical tests were applied to the data using R 4.2.0 software, and the results were drawn. The analysis used a 5% significance level (alpha error) and 80% power. It was assumed that 50% of cases were declared positive by both MRI and USG. These parameters ensured robust statistical analysis and interpretation of the study findings.

## Results

The demographic data of the study reveals that most participants experiencing shoulder joint pain were male (Table 1); the sample consists of 49 males (61%) and 31 females (39%). This suggests a higher prevalence of shoulder joint pain among males within the study population, potentially indicating gender-related differences in susceptibility or exposure to factors causing shoulder joint pain.

**Table 1:** Frequency distribution table for the given data on gender

Gender	Frequency	Percentage
Male	49	61%
Female	31	39%

The MRI findings indicate that partial tears are more common than complete tears across most rotator cuff tendons (Table 2). The highest occurrence of complete tears is in the supraspinatus tendon.

**Table 2:** Frequency distribution of tear findings through MRI MRI: magnetic resonance imaging

Findings	Category	Frequency	Percentage
MRI-supraspinatus partial tear	No	36	45
	Yes	44	55
MRI-supraspinatus complete tear	No	70	87.5
	Yes	10	12.5
MRI-infraspinatus partial tear	No	78	97.5
	Yes	2	2.5
MRI-infraspinatus complete tear	No	80	100
MRI-subscapularis partial tear	No	67	83.8
	Yes	13	16.3
MRI-subscapularis complete tear	No	78	97.5
	Yes	2	2.5

The USG findings reveal a similar trend to MRI, with partial tears more frequent than complete tears (Table 3). The USG showed no cases of complete tears in the infraspinatus tendon, while the supraspinatus and subscapularis tendons had a low incidence of complete tears.

**Table 3:** Frequency distribution of tear findings through USG USG: high-resolution ultrasound

Findings	Category	Frequency	Percentage
USG-supraspinatus partial tear	No	64	80
	Yes	16	19.5
USG-supraspinatus complete tear	No	73	91.3
	Yes	7	8.8
USG-infraspinatus partial tear	No	80	100.0
	Yes	0	0.0
USG-infraspinatus complete tear	No	80	100.0
USG-subscapularis partial tear	No	73	91.3
	Yes	7	8.8
USG-subscapularis complete tear	No	78	97.5
	Yes	2	2.5

Kappa statistics reveal varying levels of agreement between USG and MRI findings for different types of shoulder tears (Table 4). The agreement for supraspinatus partial tears is moderate (Kappa = 0.4, 62.5% agreement), whereas for supraspinatus complete tears, the agreement is almost perfect (Kappa = 0.961, 96.25% agreement). The agreement for infraspinatus partial tears is very high (Kappa = 0.974, 97.5% agreement), while for infraspinatus complete tears, the agreement is perfect, although no cases were detected. Subscapularis partial and complete tears also show high agreement levels (Kappa = 0.918, 92.5% and Kappa = 1, 100% respectively). These results indicate that USG is generally reliable in detecting complete tears but less so for partial tears, particularly for the supraspinatus muscle.

**Table 4:** Kappa statistics for agreement analysis on tear findings with USG in comparison with MRI MRI: magnetic resonance imaging; USG: high-resolution ultrasound

Tear Type	Imaging Method	Kappa Statistics	Agreement %	USG Result	MRI Yes (TP/FP)	MRI No (FN/TN)	Total
Supraspinatus partial tear	MRI	0.4	62.50%	USG Yes	15 (TP)	1 (FP)	16
				USG No	29 (FN)	35 (TN)	64
				Total	44	36	80
Supraspinatus complete tear	MRI	0.961	96.25%	USG Yes	7 (TP)	0 (FP)	7
				USG No	3 (FN)	70 (TN)	73
				Total	10	70	80
Infraspinatus partial tear	MRI	0.974	97.50%	USG Yes	0 (TP)	0 (FP)	0
				USG No	2 (FN)	78 (TN)	80
				Total	2	78	80
Infraspinatus complete tear	MRI	-	100%	USG Yes	0 (TP)	0 (FP)	0
				USG No	0 (FN)	80 (TN)	80
				Total	0	80	80
Subscapularis partial tear	MRI	0.918	92.50%	USG Yes	7 (TP)	0 (FP)	7
				USG No	6 (FN)	67 (TN)	73
				Total	13	67	80
Subscapularis complete tear	MRI	-	100%	USG Yes	2 (TP)	0 (FP)	2
				USG No	0 (FN)	78 (TN)	78
				Total	2	78	80

USG findings align with MRI in detecting shoulder conditions, with high rates of biceps tendinopathy, supraspinatus tendinitis, and subscapularis tendinopathy (Table 5).

**Table 5:** Frequency distributions of MRI findings for various shoulder conditions MRI: magnetic resonance imaging; GHL: glenohumeral ligament; ACJ DEG: acromioclavicular joint degeneration

MRI Findings		Frequency	Percentage
MRI-biceps tendinopathy	No	39	48.8
	Yes	41	51.3
MRI-Bankart lesion	No	70	87.5
	Yes	10	12.5
MRI-Hill-Sachs lesion	No	62	77.5
	Yes	18	22.5
MRI-supraspinatus tendinitis and tendinopathy	No	77	96.3
	Yes	3	3.8
MRI-supraspinatus strain	No	0	0.0
	Yes	80	100.0
MRI-subscapularis tendinopathy	No	75	93.8
	Yes	5	6.3
MRI-subscapularis strain	No	80	100.0
	Yes	0	0.0
MRI-deltoid partial tear	No	78	97.5
	Yes	2	2.5
MRI-deltoid strain	No	78	97.5
	Yes	2	2.5
MRI-impingement	No	79	98.8
	Yes	1	1.3
MRI-osteonecrosis of humerus	No	79	98.8
	Yes	1	1.3
MRI-trapezius partial tear	No	79	98.8
	Yes	1	1.3
MRI-adhesive capsulitis	No	79	98.8
	Yes	1	1.3
MRI-Perthes lesion	No	79	98.8
	Yes	1	1.3
MRI-GHL injuries	No	76	95.0
	Yes	4	5.0
MRI-coracohumeral ligament injuries	No	78	97.5
	Yes	2	2.5
MRI-R cuff interval tear	No	79	98.8
	Yes	1	1.3
MRI-ACJ DEG changes	No	62	77.5
	Yes	18	22.5
MRI-DEG changes and effusion in the shoulder joint	No	51	63.8
	Yes	29	36.3
MRI-subcoracoid and subacromial bursitis	No	73	91.3
	Yes	7	8.8

Kappa statistics demonstrate variable agreement between MRI and USG. The highest agreement is for infraspinatus complete tears and MRI findings, whereas the lowest agreement is for supraspinatus partial tears (Table 6).

**Table 6:** Frequency distributions of USG findings for various shoulder conditions USG: high-resolution ultrasound; ACJ DEG: acromioclavicular joint degeneration

USG Findings		Frequency	Percentage
USG-biceps tendinopathy	No	38	47.5
	Yes	42	52.5
	Total	80	100.0
USG-supraspinatus tendinitis and tendinopathy	No	67	83.8
	Yes	13	16.3
	Total	80	100.0
USG-subscapularis tendinopathy	No	69	86.3
	Yes	11	13.8
	Total	80	100.0
USG-ACJ DEG changes	No	58	72.5
	Yes	22	27.5
	Total	80	100.0
USG-DEG changes and effusion in the shoulder joint	No	51	63.8
	Yes	29	36.3
	Total	80	100.0

The agreement between MRI and USG is generally high, with the highest agreement for conditions such as biceps tendinopathy and degenerative changes of the acromioclavicular joint. The lowest agreement is observed for supraspinatus tendinitis and tendinopathy (Table 7).

**Table 7:** Kappa statistics for agreement analysis on shoulder findings with USG in comparison with MRI USG: high-resolution ultrasound; MRI: magnetic resonance imaging

Tear Type	Imaging Method	Kappa Statistics	Agreement %	USG Result	MRI Yes (TP/FP)	MRI No (FN/TN)	Total
Supraspinatus partial tear	MRI	0.4	62.50%	USG Yes	15 (TP)	1 (FP)	16
				USG No	29 (FN)	35 (TN)	64
				Total	44	36	80
Supraspinatus complete tear	MRI	0.961	96.25%	USG Yes	7 (TP)	0 (FP)	7
				USG No	3 (FN)	70 (TN)	73
				Total	10	70	80
Infraspinatus partial tear	MRI	0.974	97.50%	USG Yes	0 (TP)	0 (FP)	0
				USG No	2 (FN)	78 (TN)	80
				Total	2	78	80
Infraspinatus complete tear	MRI	-	100%	USG Yes	0 (TP)	0 (FP)	0
				USG No	0 (FN)	80 (TN)	80
				Total	0	80	80
Subscapularis partial tear	MRI	0.918	92.50%	USG Yes	7 (TP)	0 (FP)	7
				USG No	6 (FN)	67 (TN)	73
				Total	13	67	80
Subscapularis complete tear	MRI	-	100%	USG Yes	2 (TP)	0 (FP)	2
				USG No	0 (FN)	78 (TN)	78
				Total	2	78	80
Biceps tendinopathy	MRI	0.9875	98.70%	USG Yes	41 (TP)	1 (FP)	42
				USG No	0 (FN)	38 (TN)	38
				Total	41	39	80
Supraspinatus tendinitis and tendinopathy	MRI	0.8571	87.50%	USG Yes	3 (TP)	10 (FP)	13
				USG No	0 (FN)	67 (TN)	67
				Total	3	77	80
Subscapularis tendinopathy	MRI	0.918	92.50%	USG Yes	5 (TP)	6 (FP)	11
				USG No	0 (FN)	69 (TN)	69
				Total	5	75	80
USG-acromioclavicular joint degenerative changes	MRI	0.947	95.00%	USG Yes	18 (TP)	4 (FP)	22
				USG No	0 (FN)	58 (TN)	58
				Total	18	62	80
MRI-degenerative changes, shoulder joint effusion	MRI	100	100%	USG Yes	29 (TP)	0 (FP)	29
				USG No	0 (FN)	51 (TN)	51
				Total	29	51	80

## Discussion

This study aimed to compare the diagnostic efficacy of USG and MRI in detecting shoulder joint pathologies at a tertiary care hospital in Central India. The findings indicate that both imaging modalities have their strengths and limitations, with MRI being superior for comprehensive evaluations and USG offering a reliable, cost-effective alternative for initial assessments. Our results show that USG has high sensitivity and specificity for detecting complete rotator cuff tears, with kappa values indicating almost perfect agreement with MRI findings for supraspinatus complete tears (κ = 0.961) and subscapularis complete tears (κ = 1.0). These findings are consistent with previous studies that have demonstrated the high diagnostic accuracy of USG for complete rotator cuff tears [5,8,12]. However, USG was less sensitive in detecting partial tears, particularly for the supraspinatus muscle, where the agreement with MRI was moderate (κ = 0.4). This limitation has been noted in earlier research, which suggests that USG may miss small or subtle partial tears that are more easily detected by MRI [13].

MRI remains the gold standard for shoulder imaging due to its superior soft tissue contrast and ability to visualize complex structures. It was particularly effective in identifying partial tears and other pathologies such as biceps tendinopathy and Bankart lesions. The high prevalence of these conditions detected by MRI in our study aligns with previous literature [3,14,15]. Moreover, MRI's ability to assess the entire rotator cuff complex and associated structures makes it invaluable for comprehensive evaluations and surgical planning [3,16]. Despite its limitations, USG has several advantages that make it a valuable tool in clinical practice. It is widely available, cost-effective, and allows for dynamic assessment of shoulder structures [9,17]. The high agreement rates between USG and MRI for complete tears and several other conditions in our study support its use as a first-line imaging modality, particularly in resource-limited settings [18]. Additionally, USG's real-time imaging capability facilitates the identification of dynamic abnormalities and can guide interventions such as corticosteroid injections [19-21].

The study's setting in a rural tertiary care hospital highlights the importance of accessible and cost-effective diagnostic tools such as USG. In rural and resource-limited areas, MRI may not be readily available, and the high costs associated with MRI can be prohibitive for many patients [22,23]. USG offers a practical alternative, enabling timely diagnosis and management of shoulder pathologies, thereby improving patient outcomes [24]. There are several limitations to this study. The cross-sectional design limits the ability to assess the long-term outcomes of patients diagnosed with shoulder pathologies. The study population was also limited to patients attending a single tertiary care hospital, which may not represent the broader population. Future research should focus on longitudinal studies to evaluate the long-term efficacy of USG in diagnosing and managing shoulder pathologies. Further studies comparing the cost-effectiveness of USG and MRI in different healthcare settings would also be valuable.

Limitations

This study's limitations include a relatively small sample size of 80 patients and its single-center design, which may limit the generalizability of the findings. The cross-sectional nature of the study prevents the assessment of long-term outcomes. Additionally, the exclusion of patients with certain conditions, such as infective arthritis, rheumatoid arthritis, and previous shoulder surgeries, may restrict the applicability of the results. Operator dependency in ultrasound imaging may also introduce variability. Future research with larger, multicenter cohorts and longitudinal follow-up is needed to confirm these findings and further evaluate the diagnostic utility of ultrasound in different settings.

## Conclusions

The findings of this study underscore the significant role of USG as an effective diagnostic tool for the initial assessment of shoulder joint pain. While MRI remains the gold standard for detailed evaluation, particularly for detecting partial tears and complex pathologies, USG demonstrates substantial reliability in diagnosing complete rotator cuff tears and various shoulder conditions. The high agreement levels between USG and MRI findings, especially for complete tears, highlight USG's potential as a cost-effective, accessible, and non-invasive alternative in clinical settings, particularly in resource-limited environments. Consequently, incorporating USG into the diagnostic protocol for shoulder pain can enhance patient management by providing timely and accurate preliminary assessments, ultimately facilitating better clinical outcomes.
